# DNA methyltransferase 1 inhibits microRNA-497 and elevates GPRC5A expression to promote chemotherapy resistance and metastasis in breast cancer

**DOI:** 10.1186/s12935-022-02466-5

**Published:** 2022-03-07

**Authors:** Yaobang Liu, Zhengyang Bai, Dahai Chai, Yali Gao, Ting Li, Yinling Ma, Jinping Li

**Affiliations:** 1 Department of Surgical Oncology, General Hospital of Ningxia Medical University, No. 804, Shengli South Street, Xingqing District, Yinchuan, 750004 Ningxia People’s Republic of China; 2 Ningxia Medical University, Yinchuan, 750004 People’s Republic of China

**Keywords:** DNA methyltransferase 1, Breast cancer, MicroRNA-497, G-protein-coupled receptor family C group 5 member A, CpG island

## Abstract

**Background:**

Abnormal DNA methylation of tumor suppressor gene promoter has been found in breast cancer. Therefore, the current study set out to explore how DNA methyltransferase 1 (DNMT1) affects breast cancer through mediating miR-497/GPRC5A axis.

**Methods:**

After loss and gain-of-function approaches were conducted in MCF-7/ADR and MCF-7 cells, cell viability, IC50 value, invasion, migration and apoptosis were measured, respectively. In addition, drug resistance, metastasis and apoptosis-related protein expression were examined using immunoblotting. ChIP and dual-luciferase reporter gene assays were carried out to validate relationship among DNMT1, miR-497, and GPRC5RA. Subcutaneous xenograft tumor model in nude mice was established to detect effects of DNMT1 on growth and metastasis of breast cancer in vivo.

**Results:**

It was found that DNMT1 was notably increased, while miR-497 was poorly-expressed in breast cancer. Highly-expressed DNMT1 could promote chemotherapy resistance and metastasis of breast cancer. Meanwhile, DNMT1 modified methylation of CpG island in miR-497 promoter region, thereby repressing miR-497 level. In addition, miR-497 targeted GPRC5A expression to curb chemotherapy resistance and metastasis of breast cancer cells. Lastly, in vivo experiments showed that knockdown of DNMT1 could suppress breast cancer growth and metastasis.

**Conclusions:**

Collectively, our findings indicated that DNMT1 may inhibit miR-497 and boost the expression of GPRC5A through methylation, thus augmenting breast cancer chemotherapy resistance and metastasis, which provides novel mechanistic insight into the unrecognized roles of DNMT1 in breast cancer.

**Supplementary Information:**

The online version contains supplementary material available at 10.1186/s12935-022-02466-5.

## Background

Breast cancer is a highly-prevalent, stem cell disease featured by the presence of cancer cells with stem cell-like characteristics and the ability to potentiate tumor initiation [1]. As malignancy of the mammary glands of mammals, breast cancer is a leading cause of cancer-related deaths among women [2]. A number of factors can contribute to the high mortality rates, with metastasis to vital organs prevailing as the leading cause [3]. Currently, chemotherapy remains the gold-standard therapy for targeting invasion and metastasis in breast cancer [4]. Despite a decline in mortality due to the advent of targeted therapies and combination therapies, tumor metastasis still is attributable to considerable mortality rate [5].

DNA methyltransferase 1 (DNMT1) is well-established as a critical regulatory factor for the maintenance of epigenetic reprogramming and genome stability during DNA replication [6]. Interestingly, abnormal expressions of DNMTs and destruction of DNA methylation patterns were previously associated with carcinogenesis and cancer cell survival [7]. Moreover, a prior study uncovered the involvement of DNMT1 in several malignant tumors including breast cancer, while also exhibiting high expression levels in breast cancer [8]. It is also noteworthy that methylation of DNMT1 is significantly up-regulated in glioblastomas, whereas loss-function of DNMT1 was previously correlated with increased cell apoptosis and suppressed cell invasion [9]. DNMT1 can inhibit miR-18b-5p expression through DNA methylation, thereby promoting the proliferation of gallbladder cancer [10]. Meanwhile, existing literature further suggests that methylation-mediated mircoRNA-497 (miR-497) knockdown can promote breast cancer progression by up-regulating Mucin1 [11]. Furthermore, dysregulated microRNAs (miRNAs) are well-known to exert tumor-promoting or suppressing effects on breast cancer, whereas miR-497 was previously reported to be one of the profoundly down-regulated miRNAs in breast cancer [12]. Unsurprisingly, poor expressions of miR-497 in breast cancer patients have been associated with unfavorable clinical outcomes [13]. On a separate note, initial bioinformatic analyses in our study revealed the presence of targeting relationship between the G-protein-coupled receptor family C group 5 member A (GPRC5A) and miR-497. As a member of the GPCR superfamily, abnormal expressions of GPRC5A are associated with tumorigenesis, while GPRC5A is also significantly expressed in breast cancer [14, 15]. In light of the aforementioned evidence, we investigated how DNMT1 affects breast cancer by mediating the miR-497/GPRC5A axis.

## Methods

### Ethics statement

All human and animal experimentation protocols were approved by the Ethics Committee of General Hospital of Ningxia Medical University. Signed informed consents were obtained from all participants, and all experimental animals operating procedures were in line with the Guide for the Care and Use of Laboratory Animals published by the National Institutes of Health.

### Bioinformatics analysis

Firstly, the UALCAN database was adopted to retrieve miR-497 and DNMT1 expression in breast cancer and normal samples included in The Cancer Genome Atlas (TCGA). Additionally, breast cancer expression dataset GSE33447 was obtained through the GEO database, which comprised of 8 normal samples and 8 tumor samples. With normal samples taken as control, the R language “limma” package was adopted for differential analysis with |logFC| >  2, FDR  <  0.05 serving as screening criteria. The GEPIA database was further employed to search differentially expressed genes in breast cancer and normal samples included in TCGA and GTEx. Furthermore, the enrichment of H3K27me3 and H3K4me3 in miR-497 promoter region in MCF-7 cells were obtained from the ENCODE database. Moreover, the downstream targets of miR-497 were predicted with the help of the StarBase database.

### Collection of clinical samples

Forty two pairs of breast cancer and adjacent normal tissues were surgically collected from breast cancer female patients (age ranging between 31 and 62 years, median age: 44 years) who were hospitalized at the General Hospital of Ningxia Medical University from 2017 to 2019. All collected samples were pathologically diagnosed as breast cancer by two experienced pathologists. The enrolled patients had no other malignant tumors, and had not undergone preoperative chemotherapy or radiotherapy. Tissue specimens obtained after resection were immediately stored at − 80 °C.

### Cell culture

Human normal breast epithelial cells MCF-10A, Adriamycin (ADR) resistant breast cancer MCF-7 cells (MCF-7/ADR), and ADR sensitive breast cancer cells MCF-7 were procured from the Cell Bank of Chinese Academy of Sciences (Shanghai, China). Subsequently, MCF-10A and MCF-7 cells were cultured in RPMI-1640 medium (Gibco BRL, Grand Island, NY), while MCF-7/ADR cells were incubated in 1.0 μmol/L ADR-containing RPMI-1640 medium supplemented with 100 U/mL penicillin (Sigma-Aldrich, St. Louis), 100 mg/mL streptomycin (Sigma-Aldrich) and 10% fetal bovine serum (FBS) (Gibco BRL).

### Plasmid and lentivirus transduction

The aforementioned cells were then transfected with oe-NC, oe-DNMT1, si-NC, si-DNMT1, NC inhibitor, miR-497 inhibitor, NC mimic, miR-497 mimic, control or GPRC5A. Briefly, 1 × 10^5^ cells were inoculated in each well of a 6-well plate. Upon reaching 60–70% cell confluence, 750 μL OptiMEM culture solution was added, followed by addition of 250 μL transfection reagent and the mixture of LIPO3000 (Invitrogen, Carlsbad, CA). The solution was changed after 16 h. After 48 h, the cellular RNA content was extracted, and after 72 h, the cellular protein content was extracted for subsequent experiments.

Afterwards, the cells were treated with lentiviral vector expressing shRNA against negative control (sh-NC) or shRNA against DNMT1 (sh-DNMT1). All lentiviral vectors carried luciferase. Prior to formal experiments, the lentiviral vectors were diluted with phosphate buffer saline (PBS) into different titer gradients according to the provided virus titer, and then MCF-7/ADR cells in the 96-well plate were infected with the virus for 24 h. Fluorescence intensity of green fluorescent proteins (GFP) with different titers was observed using a fluorescence microscope. Thereafter, 5 × 10^4^ cells were seeded in a 24-well plate, and added with virus solution and 10 μg/mL Polybrene (H8761, Solarbio, Beijing, China). After 16–24 h, the solution was changed. After 72 h, 1 μg/mL purinomycin (A1113803, Invitrogen) was added for cell screening. When the cells exhibited steady growth, the down-regulation effect was detected by means of RT-qPCR. All plasmids and lentiviruses were purchased from Genechem (Shanghai, China).

### RT-qPCR

Abiding to the manufacturer’s protocols, miR Vana™ PARISTM RNA kits (AM1556, Invitrogen) was adopted for isolation of miRNA from extracellular vesicles (EVs), tissues, and cells. For qPCR of mRNA, cDNA was synthesized with the help of reverse transcription kits (M1701, Promega, Madison, WI). Afterwards, the obtained cDNA samples were warm bathed at 80 °C for 5 min to inactivate reverse transcriptase for subsequent PCR reaction. For miRNA qPCR, the primers in the kit were replaced. In the current experiment, miRNA first strand cDNA synthesis (tailing reaction) (b532451, Sangon, Shanghai, China) was adopted for reverse transcription. Finally, quantitative analysis of RNA was performed using Fast SYBR Green PCR kits (Applied Biosystems, NY) and an ABI Prism 7300 RT-qPCR system (Applied Biosystems), with three parallel wells set for each sample. U6 was employed as internal reference of miR-497 and GAPDH for other genes. 2^−ΔΔCT^ method was finally adopted to measure the target gene relative expression. All primers (Additional file 1: Table S1) were purchased from Sangon.

### Immunoblotting

After lysing with 60 μL RIPA lysate containing 1% protease inhibitor and 1% phosphorylase inhibitor (Beyotime, Shanghai, China) on ice for 45 min, cells were centrifugated at 12,000×*g* for 15 min at 4 °C and protein supernatant was obtained. Protein concentration was measured using BCA kits (Beyotime Biotechnology). A quarter volume of 5 ×  Loading Buffer (Beyotime Biotechnology) was then added to the protein solution, which was further boiled at 100 °C for 10 min. Next, 20 μg of each protein solution was taken and separated in 10% SDS-PAGE gel by electrophoresis and the isolated protein gel was transferred to a PVDF membrane. After that, membrane was placed in blocking solution containing 5% bovine serum albumin (BSA) for sealing for 2 h. Subsequently, bands of corresponding molecular segments were put into the prepared primary antibody (Additional file 1: Table S2) and incubated at 4 °C overnight (12–16 h). The bands were then placed into the prepared horseradish peroxidase (HRP)-labeled secondary antibody and incubated at ambient temperature for 1 h. Following incubation, the bands were added with the ECL working solution (BM101, Biomiga, Inc. San Diego, CA). After 1 min, development was performed using the BioSpectrum 600 Imaging System (Ultra-Violet Products, Cambridge, UK). The Image J software was adopted to calculate gray value of protein bands, and gray value of target protein/GAPDH gray value was used as relative protein expression.

### Methylation specific PCR (MSP)

With the help of DNA Methylation Gold™ kits (D5005, Zymo Research, Irvine, CA), a tube of CT conversion reagent was mixed with 900 μL water, 50 μL M-solution buffer, and 300 μL M-dilution buffer (10 min). Subsequently, 20 μL DNA sample was reacted with 130 μL prepared CT conversion reagent at 98 °C for 10 min, 64 °C for 2.5 h, and stored at 4 °C. Zymo-Spin IC column was then added with 600 μL of M-binding buffer and mixed upside down. The centrifugal force was  > 10,000*g* for 30 s. After the addition of 100 μL M-WASI buffer, the column was centrifuged (30 s), 200 μL M-desulfonation buffer was added, and allowed to stand for 15–20 min. Following repeated centrifugation (30 s), the column was added with 200 μL M-cleaning buffer, and subjected to centrifugation for 30 s. Later, 10 μL M-elution buffer was added to the column material, placed in a 1.5 mL EP and centrifuged to elute DNA.

Methylation primer sequences used for MSP amplification and non-methylation reaction are illustrated in Additional file 1: Table S3. The purified DNA was modified with bisulfite and purified, and then subjected to PCR. Sample without template were employed as NC, and the sample with CpGenome Universal Methylated DNA (Merck KGaA, Darmstadt, Germany) as template was regarded as positive control. The reaction products were subsequently subjected to agarose gel electrophoresis and photography, and then analyzed with the image analysis system.

### ChIP assay

First, 243 μL of 37% formaldehyde (final concentration: 1%) was added to cells on a 10 cm plate and incubated at 37 °C for 10 min. Subsequently, 450 μL 2.5 M glycine was added to a final concentration of 0.125 M to terminate crosslinking. Following medium removal, cells were collected in a 15 mL centrifuge tube. The sample in centrifuge tube was centrifuged (1006.2×*g*, 5 min) to collect the cells. After the supernatant was discarded, SDS lysis buffer to final concentration was 1 × 10^6^ cells/100 μL, followed by the addition of protease inhibitors. Afterwards, the cells were destroyed by ultrasonification. The DNA was broken to 300–800 bp size, and centrifuged at 10,000×*g* at 4 °C for 10 min.

Secondly, 100 μL of solution antibody containing DNMT1 (E-1001-050; dilution ratio of 1: 100, ab13537, A&D Technology Corporation, Beijing, China), H3 (ab1791, Abcam), H3K4me3 (ab8580, Abcam), and H3K27me3 (17-622, Millipore, Billerica, MA) was added to the sample, as the experimental group binding with miR-497 promoter. Subsequently, 100 μL of untreated solution was added to the sample as control group, and 4 μL of 5 M NaCl (final concentration: 0.2 M) was added to the other 100 μL of the sample, and the crosslinking was de-crosslinked (65 °C; 3 h), followed by electrophoresis. Next, 100 μL partial lysate was incubated with 900 μL of ChIP dilution buffer, 20 μL of 50 ×  PIC, and incubated with 60 μL of Protein A and agarose/salmon sperm DNA (4 °C; 1 h). Afterwards, the sample was precipitated at 4 °C for 10 min, and centrifuged for 1 min to collect the supernatant, and 20 μL of sample was retained as the input group. Among them, 1 μL of antibody was added to one tube and the other tube was untreated.

Afterwards, 100 μL destruction products were cross-linked by adding 4 μL of 5 M NaCl at 65 °C for 2 h. Next, 60 μL of Protein A and agarose/salmon sperm DNA were incubated in each tube (4 °C; 2 h). The sample was left standing (4 °C; 10 min), and then subjected to centrifugation for 1 min. After supernatant removal, the precipitated complex was centrifuged for 1 min. After supernatant removal another time, the elution buffer was prepared by mixing 100 μL 10% SDS, 100 μL 1 M NaHCO3, and 800 μL ddH_2_O (total volume: 1 mL). Then, 250 μL elution buffer was mixed upside down in test tube at ambient temperature for 15 min, and supernatant was centrifuged.

Finally, 500 μL of the elution buffer was mixed with 20 μL of 5 M NaCl (final concentration: 0.2 M), and de-crosslinked at 65 °C overnight. The following day, 1 μL RNASeA (MBI, Vilnius, Lithuania) was incubated in a tube and at 37 °C for 1 h, followed by addition of 10 μL of 0.5 M EDTA, 20 μL of L1 M Tris–HCl (pH  = 6.5), and 2 μL of 10 mg/mL protease K (45 °C; 2 h). Omega Gel was adopted to recycle test kits. In the end, the samples were dissolved in 100 μL ddH2O, followed by RT-qPCR.

### CCK-8 assay

After the cells were trypsinized and centrifuged, cell suspension was seeded in a 96-well plate (5 × 10^3^ cells). 5 parallel wells were set. After 24 h, original medium was discarded, and cells were incubated with different concentrations of ADR (diluted with PBS, the concentration gradient was set to 0, 20, 40, 60, 80, 100 μmol/L) for 48 h. Subsequently, ADR solution was discarded, and 110 μL of CCK-8 mixture solution (DMEM:CCK8 reagent  = 100 μL:10 μL) was added to each well (Dojindo Laboratories, Kumamoto, Japan). Later, the 96-well plate was cultured in a 37 °C incubator for 0.5–2 h. Afterwards, the absorbance OD value was detected at 450 nm using a microplate analyzer, and the IC50 value was calculated using the SPSS21.0 statistical software.

### Flow cytometry

Cells (1 × 10^6^ cells/well) were seeded in a 6-well plate, and collected 24 h later, followed by detachment and centrifugation. Subsequently, Annexin V-FITC/PI, 400 μL of Annexin V binding solution were added to the cell precipitates to resuspend the cells. Next, the cells were incubated with 10 μL PI and 5 μL Annexin V-FITC staining in conditions void of light at 4 °C for 10 min, and apoptosis was detected by means of FACSCalibur flow cytometry (BD Biosciences, San Jose, CA).

### Transwell assay

The chambers were pre-linked with Matrigel gel (356235, BD BioCoat Matrigel Invasion Chamber, BD Biosciences). After being cultured in serum-free DMEM for 24 h, cells were counted, and the concentration of cell suspension was adjusted to 1 × 10^5^ cells/mL. Basolateral chamber of forage hole was added with DMEM medium containing 10% FBS, while the Apical chamber was added with 200 μL cell suspension. After 48-h cell growth, uninvaded cells and Matrigel gel were carefully wiped with a cotton swab, and the Transwell chamber was taken out, fixed, and then stained with 0.5% crystal violet. Afterwards, 5 visual fields were randomly selected under an inverted microscope for observation, photography and counting.

### Scratch assay

Cells (1 × 10^6^ cells/well) were seeded in a 6-well plate and cultured at 37 °C. When cells were cultured into monolayer cells, scratches were made in the cells with pipette tips. The time of making scratches was set to 0 h, and was observed once every 12 h, with the image recorded at 48 h. The ImageJ software (National Institutes of Health, Bethesda, MD) was adopted to analyze the images and calculate the percentage of wound healing area.

### Dual-luciferase reporter gene assay

HEK-293 T cell lines (procured from the Cell bank of Shanghai Institute of Cell Research, Chinese Academy of Sciences) were cultured in DMEM. Upon 80–90% confluence, cells were detached and passaged, and then routinely cultured in an incubator in 5% CO_2_ at 37 °C. Cells were selected for experiment. The GPRC5A 3′UTR gene fragment was artificially synthesized, and then introduced into the pmirGLO vector (Promega) using endonuclease sites to design complementary sequence mutation sites on the GPRC5A wild type. The target fragment was subsequently inserted into the pGL3-control vector using T4 DNA ligase after restrictive endonuclease treatment.

Afterwards, the correctly sequenced luciferase reporter plasmid WT and MUT were co-transfected into HEK-293 T cells with miR-497 mimic, respectively. Next, the co-transfected cells were collected and lysed 48 h after transfection, and luciferase activity was detected using a Luminometer TD-20/20 detector (E5311, Promega) with Dual-Luciferase Reporter Assay System kits (Promega).

### Immunohistochemical assay

Fresh tumor tissue specimens were sliced into appropriate sizes and fixed, dehydrated with ethanol, cut into 4-μm thickness sections, and embedded in paraffin. The paraffin sections of nude mouse tumor tissues were placed in an incubator and stored (60 °C; 2 h). Subsequently, the sections were hydrated with ethanol (100, 95, 85, 70%) and deionized water, and then immersed in citric acid buffer (0.01 mol/L, pH 6.0) and heated at 95–100 °C for 30 min. Next, the sections were incubated with 0.5% Triton  × 100 for 30 min. Afterwards, the sections were stained with a biotin-streptavidin HRP detection system (Beijing ZSGB Company, China).

The above-mentioned sections were mixed with corresponding primary antibody and incubated overnight at 4 °C. The following day, the sections were incubated with secondary antibody for 1 h, and observed under a microscope. Brown staining was indicative of a positive immune response. The immunostaining intensity was calculated according to previously stated methods [16]. The images were observed under Nikon Eclipse Ti microscope (Fukasawa, Japan) system and analyzed using the Nikon software.

### Hematoxylin–eosin (HE) staining

Tumor tissues were fixed. Next, the sections were dewaxed with xylene for 15 min and immersed in a mixture of xylene and alcohol (1:1) for 3 min, and then immersed with ethanol (100%, 95%, 90%, 80%, 70%, and 50% concentration) for 2 min respectively. After that, the tissues were stained with hematoxylin for 5 min, 1% hydrochloric acid for 2 s, then hydrated with gradient ethanol (100%, 95%, 90%, 80%) for 2 min successively, and further stained with eosin for 5 s and transparentized with xylene (15 min × 2 times). Afterwards, the sections were fixed and observed under a bright field microscope.

### Xenograft tumors in nude mice

A total of 24 male BALB/c nude mice (age range 4–6 weeks old, weight range 17–22 g) were purchased from Hunan SJA Laboratory Animal Co., Ltd. (Hunan, China). After a 1-week period of adaptive feeding, the mice were randomly divided into 4 groups (n  = 6), and placed in a specific environment with 28 °C, 50% humidity, and asepsis for regular observation. Two groups of nude mice were selected for subcutaneous tumor formation experiments. Briefly, 150 μL of MCF-7/ADR cells (2 × 10^6^) transduced with sh-DNMT1 or sh-NC were injected subcutaneously into the nude mice through the back. After 1 week, the mice were injected with ADR 0.1 mL (25 mg/kg, intraperitoneal injection), twice a week. Tumor growth in nude mice was monitored weekly, and tumor volume (maximum diameter  ×  minimum diameter^2^ × 0.5) was calculated. After 6 weeks of medication, the mice were anesthetized by means of cervical dislocation. The transplanted tumor was then separated from the nude mice, and the tumor weight was immediately measured. The obtained tumor tissues were fixed to make pathological sections, which were fixed with 4% paraformaldehyde, and the expression patterns of various factors were detected by RT-qPCR and immunohistochemical.

MCF-7/ADR cells (2 × 10^6^ cells) transduced with sh-DNMT1 or sh-NC were injected into nude mice (150 μL) via tail vein, followed by the ADR treatment. After 5 weeks, the nude mice were anesthetized with phenobarbital sodium and fluorescence imaging was performed in vivo using the LB983 NIGHTOWL II system (Berthold Technologies GmbH, Calmbacher, Germany), and lung metastases were observed.

### Statistical analysis

Measurement data, summarized by mean  ±  standard deviation, were analyzed using SPSS 21.0 software (IBM, Armonk, New York), with statistical significance at *p*  < 0.05. Data comparison between tumor tissues and adjacent normal tissues was conducted by means of paired *t* test, while unpaired *t* test was adopted for comparisons of unpaired data between two groups. One-way ANOVA was conducted for multiple group comparison, followed by Tukey’s post-hoc test. Two-way ANOVA was performed to compare data between two groups at different time points, and the IC50 value was analyzed after log transformation. Pearson correlation analysis was adopted to analyze correlation between indicators.

## Results

### DNMT1 participates in the occurrence of breast cancer by mediating miR-497 methylation

**Fig. 1:**
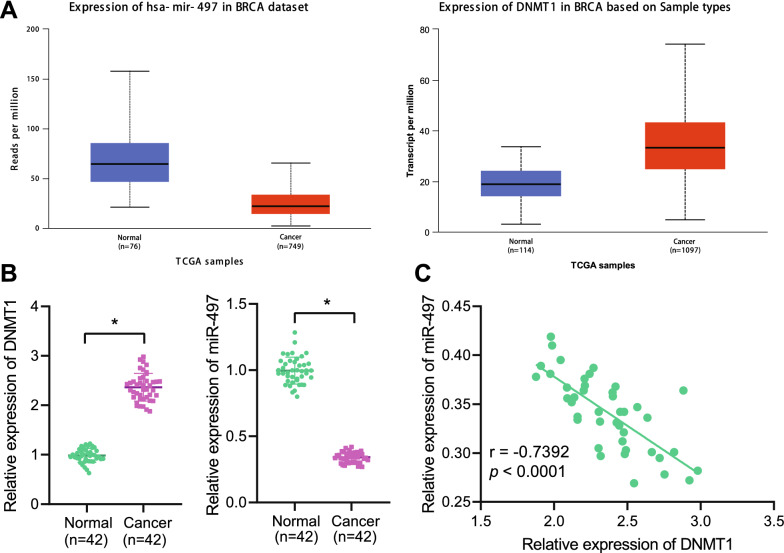
Expression of DNMT1 and miR-497 in breast cancer tissues was examined. **A** The expression of DNMT1 in 1097 breast cancer samples and 114 adjacent normal tissue samples (left), and expression of miR-497 in 749 breast cancer samples and 76 adjacent normal tissue samples (right) collected in TCGA database. The x-axis represented sample type, the y-axis expression value, and the blue box diagram normal samples. Normal samples were shown in blue box and tumor samples shown in red box. **B** The expression of DNMT1 and miR-497 in breast cancer tissues and adjacent normal tissues from 42 patients with breast cancer detected by RT-qPCR. C, Pearson correlation analysis adopted to detect the correlation between DNMT1 and miR-497 expression in breast cancer tissues. *p  < 0.05 vs. adjacent normal tissues

Existing literature suggests that DNA methyltransferase DNMT1 possesses the ability to inhibit miR-18b-5p expression through DNA methylation, thereby promoting the proliferation of gallbladder cancer. Apart from that, DNMT1 is also known to mediate the methylation of miR-34a promoter to enhance chemoresistance of pancreatic cancer cells [17, 18]. Accordingly, we first analyzed the expression patterns of miR-497 and DNMT1 in breast cancer and adjacent normal tissues collected by TCGA through the UALCAN database, and found that miR-497 was poorly-expressed and DNMT1 was highly-expressed in breast cancer relative to adjacent normal tissues (Fig. 1A).

Furthermore, RT-qPCR illustrated suppression of miR-497 and elevation of DNMT1 in breast cancer tissues when compared to adjacent normal tissues (Fig. 1B). In addition, Pearson correlation analysis revealed that miR-497 and DNMT1 expressions were negatively-correlated (Fig. 1C).

### DNMT1 promotes drug resistance and metastasis of breast cancer

**Fig. 2:**
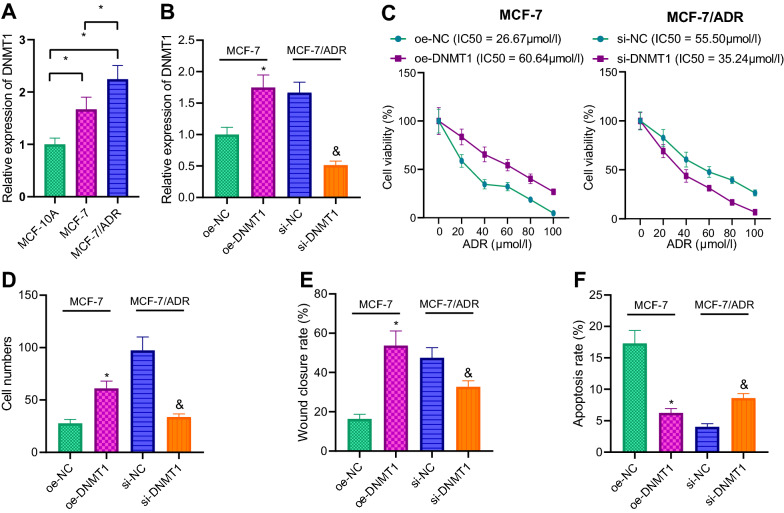
Effect of DNMT1 on chemotherapy resistance and metastasis of breast cancer cells was examined. **A** DNMT1 expression in MCF-10A, MCF-7/ADR and MCF-7 cells evaluated using RT-qPCR. **B** Transduction efficiency in si-DNMT1 or oe-DNMT1-transduced cells determined using RT-qPCR. **C** The cell viability and IC50 value of ADR detected by CCK-8 assay. **D** Cell invasion ability detected by Transwell assay. **E** The migration ability of cells detected by scratch test, and the ratio of scratch area between 48 and 0 h used as the scratch healing rate. **F** Apoptosis in oe-DNMT1-transduced MCF-7 cells and in si-DNMT1-transduced MCF-7/ADR cells examined using flow cytometry. *p  < 0.05 vs. Oe-NC; ^&^*p*  < 0.05 vs. si-NC. The experiment was repeated for 3 times independently

To further elucidate the regulatory effect of DNMT1 on breast cancer, we employed RT-qPCR and found that relative to MCF-10A cells, DNMT1 expression levels were strikingly increased in MCF-7/ADR and MCF-7 cells. Moreover, DNMT1 expression was enhanced in MCF-7/ADR cells relative to MCF-7 cells (Fig. 2A). In addition, we observed that DNMT1 was up-regulated in MCF-7 cells as a result of oe-DNMT1 transduction, while being down-regulated in MCF-7/ADR cells following si-DNMT1. Moreover, RT-qPCR validated the successful transduction in MCF-7/ADR cells and MCF-7 cells (Fig. 2B).

Furthermore, detection results of CCK-8 illustrated that the cell viability and IC50 value of ADR were both notably increased of MCF-7 cells in response to oe-DNMT1, whereas opposing trends were noted after si-DNMT1 treatment (Fig. 2C). Meanwhile, as expounded by Transwell assay and scratch assay results, oe-DNMT1 transduction resulted in increased migration and invasion abilities. On the other hand, si-DNMT1 transduction brought about reduced migration and invasion abilities (Fig. 2D, E; Additional file 1: Fig. S1A). Flow cytometric results further revealed that apoptosis was reduced in oe-DNMT1-transduced MCF-7 cells, while increased apoptosis was observed in si-DNMT1-transduced MCF-7/ADR cells (Fig. 2F; Additional file 1: Fig. S1B). In lieu of these findings, DNMT1 was believed to promote chemotherapy resistance and metastasis of breast cancer cells.

### DNMT1 promotes methylation of miR-497 promoter CpG island

**Fig. 3:**
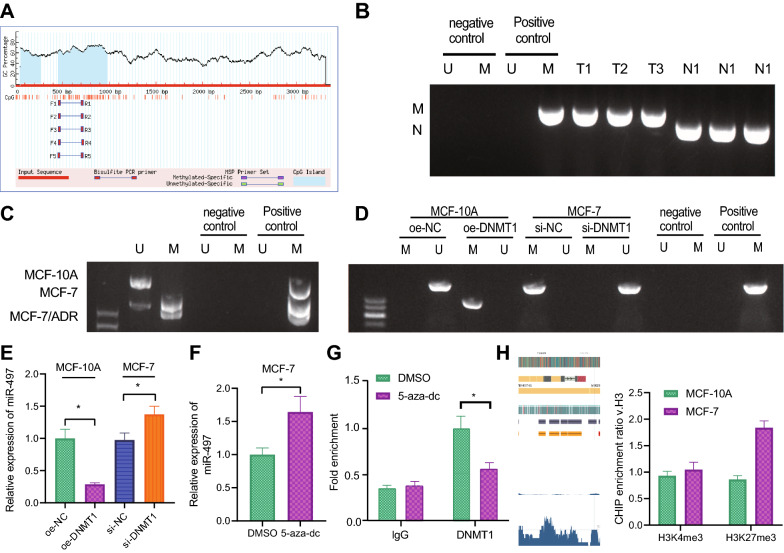
Regulation of DNMT1 on miR-497 methylation. **A** The CpG island in the promoter region of miR-497 predicted by MethPrimer website. **B** The promoter methylation status in 3 cases of breast cancer tissues and adjacent normal tissues detected using MSP. M was the methylation status and U the non-methylation status. **C** Promoter methylation status in MCF-10A, MCF-7 and MCF-7/ADR cells monitored using MSP. **D** The methylation status of miR-497 promoter in oe-DNMT1-transduced MCF-10A cells or si-DNMT1-transduced MCF-7 cells examined by MSP. **E** The expression of miR-497 in oe-DNMT1-transduced MCF-10A cells or si-DNMT1-transduced MCF-7 cells determined using RT-qPCR. **F** The expression of miR-497 after the treatment of 5-aza-dc detected using RT-qPCR. **G** The degree of enrichment of DNMT1 on the miR-497 promoter after MCF-7 cells were treated with 5-aza-dc observed by ChIP assay. **H** The enrichment of H3K27me3 and H3K4me3 in miR-497 promoter region predicted by ENCODE database and validated by ChIP assay in MCF-7 cells. *p  < 0.05 between two groups. The experiment was repeated for 3 times independently

Prediction results from the MethPrimer database illustrated the presence of CpG islands in promoter region of miR-497 (Fig. 3A). Additionally, existing evidence suggests that CpG island methylation in promoter region of miR-497 can inhibit its expression [11, 12].

Accordingly, to clarify whether DNMT1 affected miR-497 through methylation regulation, we performed a series of MSP experiments, and observed methylation of CpG island in the miR-497 promoter in cancer tissues, while there was no methylation in normal adjacent tissues (Fig. 3B). Furthermore, we also found methylation of the miR-497 promoter CpG island in MCF-7 and MCF-7/ADR cells, but no methylation was observed in MCF-10A cells (Fig. 3C).

Subsequently, we transfected MCF-10A cells with oe-DNMT1, while MCF-7 cells were treated with si-DNMT1. MSP displayed that the CpG island of miR-497 promoter exhibited methylation state in oe-DNMT1-transduced MCF-10A cells. On the other hand, si-DNMT1 transduction in MCF-7 cells brought about attenuated or eliminated CpG island methylation of miR-497 promoter (Fig. 3D). Moreover, RT-qPCR results illustrated that oe-DNMT1 transduction resulted in silenced miR-497 expression, while opposing trends were noted in response to si-DNMT1 (Fig. 3E).

Furthermore, we treated MCF-7 cells with DNA methyltransferase inhibitor 5-aza-dc to inhibit the methylation regulation of DNMT1, and found that miR-497 expression levels were boosted in MCF-7 cells treated with 5-aza-dc compared to those treated with DMSO (Fig. 3F). Meanwhile, the results of the ChIP assay showed that 5-aza-dc transduction reduced the recruitment of DNMT1 to the miR-497 promoter region compared to DMSO (Fig. 3G). Furthermore, the enrichment of H3K27me3 and H3K4me3 in miR-497 promoter region in MCF-7 cells were predicted using the ENCODE database, the results of which showed increased enrichment of H3K27me3 modification in promoter region of miR-497, in addition to insignificant enrichment of H3K4me3 modification (Fig. 3H). Moreover, ChIP assay results also validated that the enrichment of H3K27me3 on the miR-497 promoter in MCF-7 cells was increased compared to that in MCF-10A cells (Fig. 3H).

### DNMT1 inhibits miR-497 expression through methylation modification to promote breast cancer chemotherapy resistance and metastasis

**Fig. 4:**
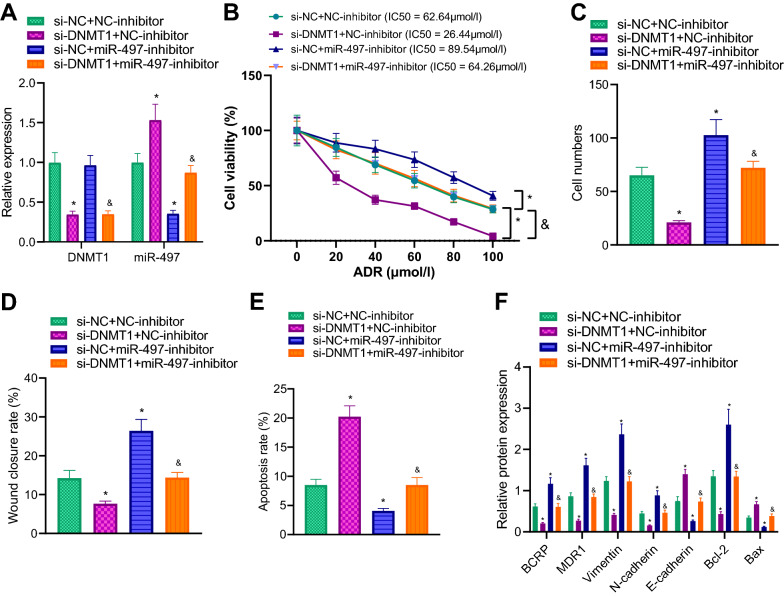
DNMT1 regulates the expression of miR-497 through methylation modification and affects breast cancer chemotherapy resistance and metastasis. **A** The expression of DNMT1 and miR-497 in MCF-7/ADR cells after transduction monitored by RT-qPCR. **B** The cell viability and IC50 value in MCF-7/ADR cells measured using CCK-8. **C** Cell invasion of MCF-7/ADR cells detected by means of Transwell assay. **D** Cell migration of MCF-7/ADR cells examined using scratch assay and the ratio of the scratch area between 48 and 0 h used as the scratch healing rate. **E** Apoptosis of MCF-7/ADR cells detected using flow cytometry. **F** Drug resistance, apoptosis and EMT-related protein expression of MCF-7/ADR cells measured by immunoblotting. *p  < 0.05 vs. MCF-7/ADR cells co-transfected with si-NC and NC-inhibitor. ^&^*p * < 0.05 vs. MCF-7/ADR cells co-transfected with si-DNMT1 and NC-inhibitor. The experiment was repeated for 3 times independently

Furthermore, we adopted a recovery experiment in MCF-7/ADR cells by transduction with the corresponding plasmids, and observed that DNMT1 expression was notably silenced whereas miR-497 expression was raised in MCF-7/ADR cells following treatment with si-DNMT1. In addition, miR-497 expression was suppressed in cells transfected with miR-497-inhibitor alone. Meanwhile, miR-497 expression was notably reduced and those of DNMT1 showed no significant changes in response to si-DNMT1 and miR-497-inhibitor (Fig. 4A).

In addition, it was found that cell viability, IC50 value, cell migration and invasion abilities of MCF-7/ADR cells were all reduced, while apoptosis was boosted in cells in the presence of si-DNMT1. Meanwhile, miR-497-inhibitor brought about enhanced cell viability, migration, invasion abilities and improved IC50 value as well as diminished apoptosis in MCF-7/ADR cells. On the other hand, miR-497-inhibition reversed the aforementioned trends of si-DNMT1 on the biological process of cells (Fig. 4B–E; Additional file 1: Fig. S1C, D).

Moreover, immunoblotting was adopted to detect drug resistance, apoptosis and EMT-related protein expression patterns, the results of which were consistent with those of cell function experiments. It was observed that si-DNMT1 transduction brought about down-regulated BCRP, MDR1, Bcl-2, Vimentin, and N-cadherin expression levels and up-regulated those of Bax and E-cadherin, while opposing trends were observed after down-regulation of miR-497. Meanwhile, simultaneous down-regulation of DNMT1 and miR-497 reversed the aforementioned effects of DNMT1 knockdown on these proteins (Fig. 4F). Altogether, these findings indicated that DNMT1 inhibited the expression of miR-497 through methylation modification, thereby promoting breast cancer chemotherapy resistance and metastasis.

### miR-497 inhibits breast cancer chemotherapy resistance and metastasis by down-regulating GPRC5A

**Fig. 5:**
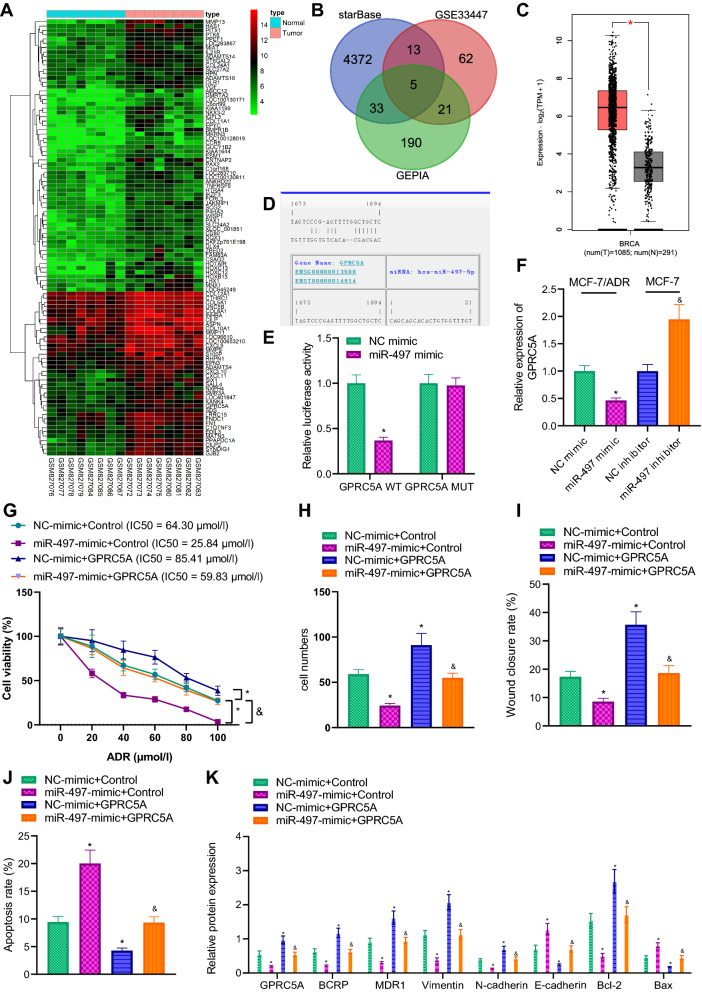
miR-497 targets and downregulates GPRC5A to affect breast cancer chemotherapy resistance and metastasis. **A** Heat map of up-regulated gene expression in breast cancer dataset GSE33447. The abscissa represented the sample number, the ordinate the gene name, and the histogram on the upper right was the color scale. **B** The intersection of miR-497 target genes predicted by StarBase database, markedly up-regulated gene from GSE33447 dataset and up-regulated genes in breast cancer from GEPIA. The middle part represented the intersection of three sets of data. **C** The differential expression of GPRC5A in breast cancer and normal samples included in TCGA and GTEx. The red box plots in the figure represented tumor samples, and the gray box plots normal samples. **D** The binding site of miR-497 and GPRC5A predicted using StarBase database. **E** The binding of miR-497 and GPRC5A verified using dual-luciferase reporter gene assay. *p  < 0.05 vs. NC-mimic group. **F** The expression of GPRC5A after up-regulation of miR-497 in MCF-7/ADR cells or down-regulation of miR-497 in MCF-7 cells detected using RT-qPCR. *p  < 0.05 vs. NC-mimic-transduced MCF-7/ADR cells. ^&^*p*  < 0.05 vs. NC-inhibitor-transduced MCF-7/ADR cells. **G** The viability and IC50 value measured using CCK-8. **H** Cell invasion detected by means of Transwell assay. I Cell migration examined using scratch assay and the ratio of the scratch area between 48 and 0 h used as the scratch healing rate. **J** Apoptosis detected using flow cytometry. **K** Drug resistance, apoptosis and EMT-related protein expression in each group of cells measured by immunoblotting. **H**–**K** The experiment was repeated for 3 times independently. *p  < 0.05 vs. NC-mimic  +  control. ^&^*p*  < 0.05 vs. miR-497-mimic  +  control

Differential analyses of the breast cancer sample dataset GSE33447 retrieved from the GEO database revealed a total of 101 highly-expressed genes in breast cancer (Fig. 5A). Subsequently, the target genes of miR-497 were predicted with the StarBase database were combined with the microarray analysis results. The intersection was obtained following GEPIA website analysis, and finally 5 candidate genes CXCL10, HOXC13, GPRC5A, MMP11, and OLR1 were identified (Fig. 5B). The expression patterns of these 5 genes in breast cancer samples were analyzed with TCGA and GTEx, which revealed that GPRC5A exhibited the highest expression in breast cancer (Fig. 5C). Moreover, existing evidence suggests GPRC5A is up-regulated in tumors and further associated with tumor metastasis [19]. Accordingly, GPRC5A was chosen as the gene of interest for follow-up research.

Afterwards, targeted binding of miR-497 to GPRC5A was predicted using the StarBase website (Fig. 5D). Subsequent results showed that luciferase activity of wild-type GPRC5A-3′UTR was decreased after transfection with miR-497-mimic (Fig. 5E). Meanwhile, RT-qPCR results illustrated that GPRC5A expression was reduced after miR-497 over-expression, while being increased following miR-497 silencing (Fig. 5F).

Furthermore, we conducted CCK-8, flow cytometry, Transwell and scratch assays in MCF-7/ADR cells to validate whether miR-497 affected breast cancer progression by regulating the GPRC5A expression. Subsequent results illustrated that transfection of miR-497-mimic brought about reduced cell viability, IC50 value, cell invasion and migration but increased apoptosis in MCF-7/ADR cells. In addition, the ectopically expressed GPRC5A resulted in increased cell viability, IC50 value, cell invasion and migration but reduced apoptosis. Meanwhile, transduction of miR-497-mimic and GPRC5A led to elevated cell viability, IC50 value, cell invasion and migration but diminished apoptosis in comparison with those treated with miR-497-mimic and control (Fig. 5G–J; Additional file 1: Fig. S1E, F). Immunoblotting further illustrated that up-regulation of miR-497 expression resulted in reduced GPRC5A, BCRP, MDR1, Bcl-2, Vimentin, and N-cadherin expression levels as well as increased Bax and E-cadherin expressions. Collectively, GPRC5A elevation could reverse the effect of miR-497 on related proteins (Fig. 5K).

### Down-regulation of DNMT1 inhibits breast cancer cell growth and metastasis in nude mice

**Fig. 6:**
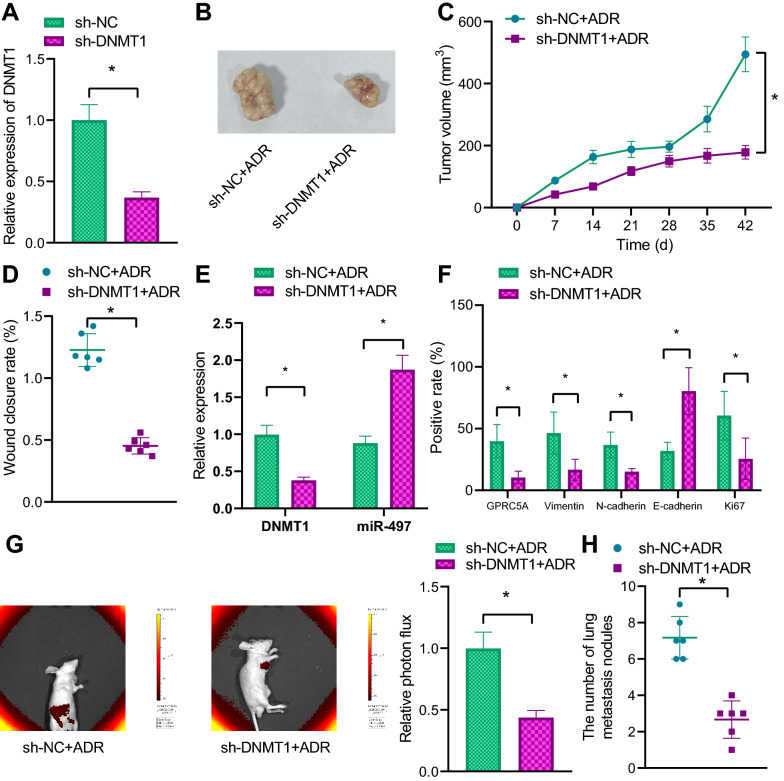
Effects of DNMT1 silencing on the growth and metastasis of breast cancer cells in nude mice. **A** The efficiency of stable knockdown of DNMT1 in MCF-7/ADR cells detected using RT-qPCR. **B** Images taken of two groups of nude mice subcutaneously transplanted tumors. **C** Line graph of tumor volume changes in nude mice, measured once a week. **D** The weight of tumor tissues in nude mice. **E** The expression of DNMT1 and miR-497 in tumor tissues of nude mice detected using RT-qPCR. **F** The expression of related proteins in tumor tissues of nude mice measured using immunohistochemical assay. **G** Tumor metastasis in nude mice evaluated using in vivo fluorescence imaging. **H** The dissected lung tissues of nude mice to observe the number of lung metastases. *p  < 0.05 vs. Tissues co-transfected with sh-NC and ADR. n  = 6

Stably transduced cells with sh-DNMT1 were successfully validated by means of RT-qPCR (Fig. 6A). The transduced cell suspension was then subcutaneously injected into nude mice, and injected with ADR 1 week later. The tumor volume and weight in the mice transduced with sh-DNMT1 were reduced in the presence of ADR (Fig. 6B–D).

Furthermore, the tumor tissues were dissected and RT-qPCR expounded that DNMT1 expression levels were reduced, while those of miR-497 were increased in tumor tissues in the presence of sh-DNMT1 and ADR (Fig. 6E). As displayed by the results of immunohistochemical assay, the levels of GPRC5A, Vimentin, N-cadherin, and Ki67 were lower in tissues in response to sh-DNMT1 and ADR relative to those to sh-NC and ADR, while E-cadherin levels were remarkably elevated (Fig. 6F).

Lastly, fluorescence intensity was reduced in mice transduced with sh-DNMT1 in the presence of ADR (Fig. 6G). As observed by HE staining, the number of metastatic nodules in the lung tissues was reduced after down-regulation of DNMT1 (Fig. 6H).

## Discussion

The process of DNA methylation is controlled by the DNMT protein to regulate the chromatin structure and gene expression of the entire genome, and further regarded as a critical epigenetic marker [20]. It is also noteworthy that aberrant over-expression of DNMT1 has been documented in various malignancies, which highlights the significant impact on cancer-related epigenetic silencing of tumor suppressor genes [21]. The obtained findings of our work revealed that DNMT1 may inhibit miR-497 expression and boost expression of GPRC5A through DNA methylation, thus augmenting chemotherapy resistance and metastasis in breast cancer.

Firstly, DNMT1 was highly-expressed and miR-497 was poorly-expressed in breast cancer, whilst exhibiting negative-correlation. Similarly, a number of studies have come across up-regulated levels of DNMT1 in TNBC and inflammatory interstitial breast cancer, in addition to over-expression of DNMT1 in the mammary gland tissues of mammary gland-specific Kindlin-2 transgenic mice [22, 23]. Interestingly, loss- function assay of miR-497, another key topic of interest in our study, was previously associated with poor prognoses of breast cancer patients [13]. On the other hand, a prior study further indicated that over-expression of miR-497 can inhibit aggressive tumor biology, and cause G1 blockade [24]. The regulatory relationship between DNMT1 and miR-497 has also been previously explored, with results suggesting that DNMT1 mediates the methylation of miR-34a promoter and promotes the resistance of pancreatic cancer cells to molecular targeted drugs [18]. Moreover, DNA methylation is known to play a significant role in regulating miR-497 in hepatocellular carcinoma [25]. Additionally, our findings further revealed iDNMT1 could promote chemotherapy resistance and metastasis of breast cancer cells, as evidenced by decreased IC50 value of ADR, diminished cell invasion and migration along with decreased apoptosis as a result of DNMT1 knockdown. In accordance with our findings, another prior study noted that down-regulation of DNMT1 exerted a suppressive effect on cell migration and invasion [26]. Besides, DNA methylation inhibition is known to repress the growth of spontaneous breast tumors in genetically engineered mouse breast cancer models, and further lead to early tumor cell necrosis and apoptosis, which in also in line our findings [27]. Moreover, we also uncovered that DNMT1 promoted CpG island methylation of the miR-497 promoter. Unsurprisingly, down-regulation miR-497 and hypermethylation at its promoter CpG island is a common occurrence in breast cancer [11]. Together, these findings and evidences suggest that DNMT1 could promote the methylation of the CpG island in miR-497 promoter region, thereby inhibiting miR-497, while the treatment of 5-aza-dc prevents methylation regulation of miR-497 by DNMT1.

Furthermore, our findings revealed that down-regulation of DNMT1 contributed to decreased BCRP, MDR1, Bcl-2, Vimentin and N-cadherin expression levels, while enhancing those of Bax and E-cadherin. These findings are particularly in line a prior study, wherein DNMT3A and DNMT3B were illustrated to repress E-cadherin expression by methylation of the E-cadherin promoter, while down-regulation of E-cadherin was associated with deterioration of DNMT3B in hospitalized breast cancer patients [28]. Above all, we uncovered that DNMT1 promoted chemotherapy resistance and metastasis of breast cancer via inhibition of miR-497 expression through methylation modification. Moreover, a prior study documented notably increased expressions of GPRC5A in breast cancer [15]. On a separate note, inhibition of miR-497 can accelerate carcinogenesis of tumors of breast cancer, whereas elevation of miR-497 is known to suppress the oncogenic phenotype of MCF-7 cells [13, 29]. Meanwhile, in gastric cancer, up-regulation of miR-497-5p was previously shown to induce inhibited cell migration, invasion and EMT [30]. Lastly, our findings revealed that down-regulation of DNMT1 inhibited growth and metastasis of breast cancer cells in nude mice. Similarly, physcion 8-*O*-β-glucopyranoside inhibits breast cancer metastasis by silencing DNMT1, which is line with our findings [26].

## Conclusions

**Fig. 7:**
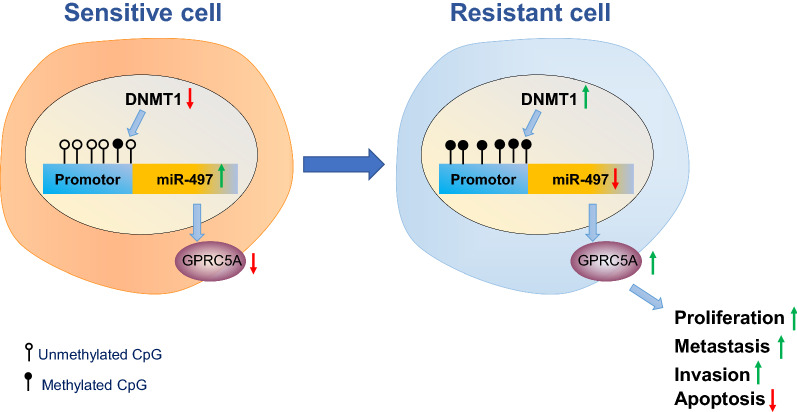
The molecular mechanism of DNA methyltransferase DNMT1 mediating miR-497/GPRC5A axis and affecting breast cancer chemotherapy resistance and metastasis

Altogether, DNMT1 inhibits miR-497 and elevates GPRC5A expression through methylation to promote chemotherapy resistance and metastasis in breast cancer (Fig. 7), which sheds a new light on the anti-tumor significance of DNMT1 in regard to breast cancer. Nevertheless, unpredictable factors require further exploration in clinical settings, while, inclusion of another breast cell line would also further strengthen our findings.

## Supplementary Information

**Additional file 1: Figure S1.** Representative images for Transwell assay and flow cytometry. Cell invasion and apoptosis in response to oe-DNMT1, as detected by means of Transwell assay (**A**) and flow cytometry (**B**). Cell invasion and apoptosis in response to si-DNMT1 and miR-497 inhibitor alone or in combination, as detected by means of Transwell assay (**C**) and flow cytometry (**D**). Cell invasion and apoptosis in response to miR-497 mimic and GPRC5A alone or in combination, as detected by means of Transwell assay (**E**) and flow cytometry (**F**). **Table S1. **Primer sequences for RT-qPCR. **Table S2.** Antibody information. **Table S3. **methylation primer sequences used for MSP amplification and non-methylation reaction.

## Data Availability

The datasets generated/analysed during the current study are available.

## Abbreviations

DNMT1: DNA methyltransferase 1
miR-497: MircoRNA-497
miRNAs: MicroRNAs
GPRC5A: G-protein-coupled receptor family C group 5 member A
TCGA: The Cancer Genome Atlas
GEO: Gene expression omnibus
ADR: Adriamycin
FBS: Fetal bovine serum
sh-DNMT1: ShRNA against DNMT1
sh-NC: ShRNA against negative control
PBS: Phosphate buffer saline
GFP: Green fluorescent proteins
RT-qPCR: Real-time quantitative polymerase chain reaction
EVs: Extracellular vesicles
GAPDH: Glyceraldehyde-3-phosphate dehydrogenase
RIPA: Radioimmunoprecipitation assay
SDS-PAGE: Sodium dodecyl sulfate polyacrylamide gel electrophoresis
PVDF: Polyvinylidene fluoride
BSA: Bovine serum albumin
TBST: Tris buffered saline tween
HRP: Horseradish peroxidase
MSP: Methylation specific PCR
ChIP: Chromatin immunoprecipitation
CCK-8: Cell counting kit 8
FITC/PI: V-fluorescein isothiocyanate/propidium iodide
UTR: Untranslated region
HE: Hematoxylin–eosin
ANOVA: Analysis of variance
