# Supplementary material for: DNA methyltransferase 1 inhibits microRNA-497 and elevates GPRC5A expression to promote chemotherapy resistance and metastasis in breast cancer
Source: Cancer Cell Int. 2022 Mar 7;22:112. doi: 10.1186/s12935-022-02466-5 (PMC8903738; doi:10.1186/s12935-022-02466-5)
Supplement: Supplementary file 1 — Additional file 1: Figure S1. Representative images for Transwell assay and flow cytometry. Cell invasion and apoptosis in response to oe-DNMT1, as detected by means of Transwell assay (A) and flow cytometry (B). Cell invasion and apoptosis in response to si-DNMT1 and miR-497 inhibitor alone or in combination, as detected by means of Transwell assay (C) and flow cytometry (D). Cell invasion and apoptosis in response to miR-497 mimic and GPRC5A alone or in combination, as detected by means of Transwell assay (E) and flow cytometry (F). Table S1. Primer sequences for RT-qPCR. Table S2. Antibody information. Table S3. methylation primer sequences used for MSP amplification and non-methylation reaction. [file 12935_2022_2466_MOESM1_ESM.docx]

**Table S1.** Primer sequences for RT-qPCR

| Gene | Primer sequences |
| --- | --- |
| miR-497 | F: 5’-CAGCAGCACACTGTGGTTTGT-3’ |
|  | R: universal reverse primer |
| DNMT1 | F: 5’-GAGGAAGCTGCTAAGGACTAGTTC-3’ |
|  | R: 5’-ACTGCACAATTTGATCACTAAATC-3’ |
| GPRC5A | F: 5’-AGACAGGGGACACGCTCTAT-3’ |
|  | R: 5’-GGAGGCAAACTGTTCCCGTA-3’ |
| U6 | F: 5’-GGGCAGGAAGAGGGCCTAT-3’ |
|  | R: universal reverse primer |
| GAPDH | F: 5’-GATTCCACCCATGGCAAATTCC-3’ |
|  | R: 5’-TCGCTCCTGGAAGATGGTGAT-3’ |

Note: miR-497, microRNA-497; DNMT1, DNA methyltransferase DNMT1; U6, U6 snRNA; GAPDH, Glyceraldehyde-3-phosphate dehydrogenase; F, forward; R, reverse.

**Table S2.** Antibody information

| Name | Molecular weight | Dilution ratio | Purchase source and cat number |
| --- | --- | --- | --- |
| BCRP | 65kDa | 0.736111111 | CST, #42078 |
| MDR1 | 130kDa | 0.736111111 | CST, #13342 |
| Vimentin | 57kDa | 0.736111111 | CST, #5741 |
| N-cadherin | 140kDa | 0.736111111 | CST, #13116 |
| E-cadherin | 135kDa | 0.736111111 | CST, #14472 |
| Bax | 20kDa | 0.736111111 | CST, #89477 |
| Bcl-2 | 26kDa | 0.736111111 | CST, #15071 |
| GPRC5A | 40kDa | 0.736111111 | CST, #12968 |
| GAPDH | 37kDa | 0.736111111 | CST, #2118 |
| Anti-rabbit IgG  (secondary antibody) | - | 0.736111111 | CST, #7074 |
| Anti-mouse IgG  (secondary antibody) | - | 0.736111111 | CST, #7076 |

**Table S3.** methylation primer sequences used for MSP amplification and non-methylation reaction

|  | Primer sequences | amplified length |
| --- | --- | --- |
| miR-497-MF | 5'-ATAAGGACGGGGATATATATATCGT-3' | 220 bp |
| miR-497-MR | 5'-AAACTACTTCCTTTACCTAAAACGC-3' |  |
| miR-497-UF | 5'-ATAAGGATGGGGATATATATATTGT-3' | 205 bp |
| miR-497-UR | 5'-AACTACTTCCTTTACCTAAAACACC-3' |  |

Note: miR-497, microRNA-497.

**
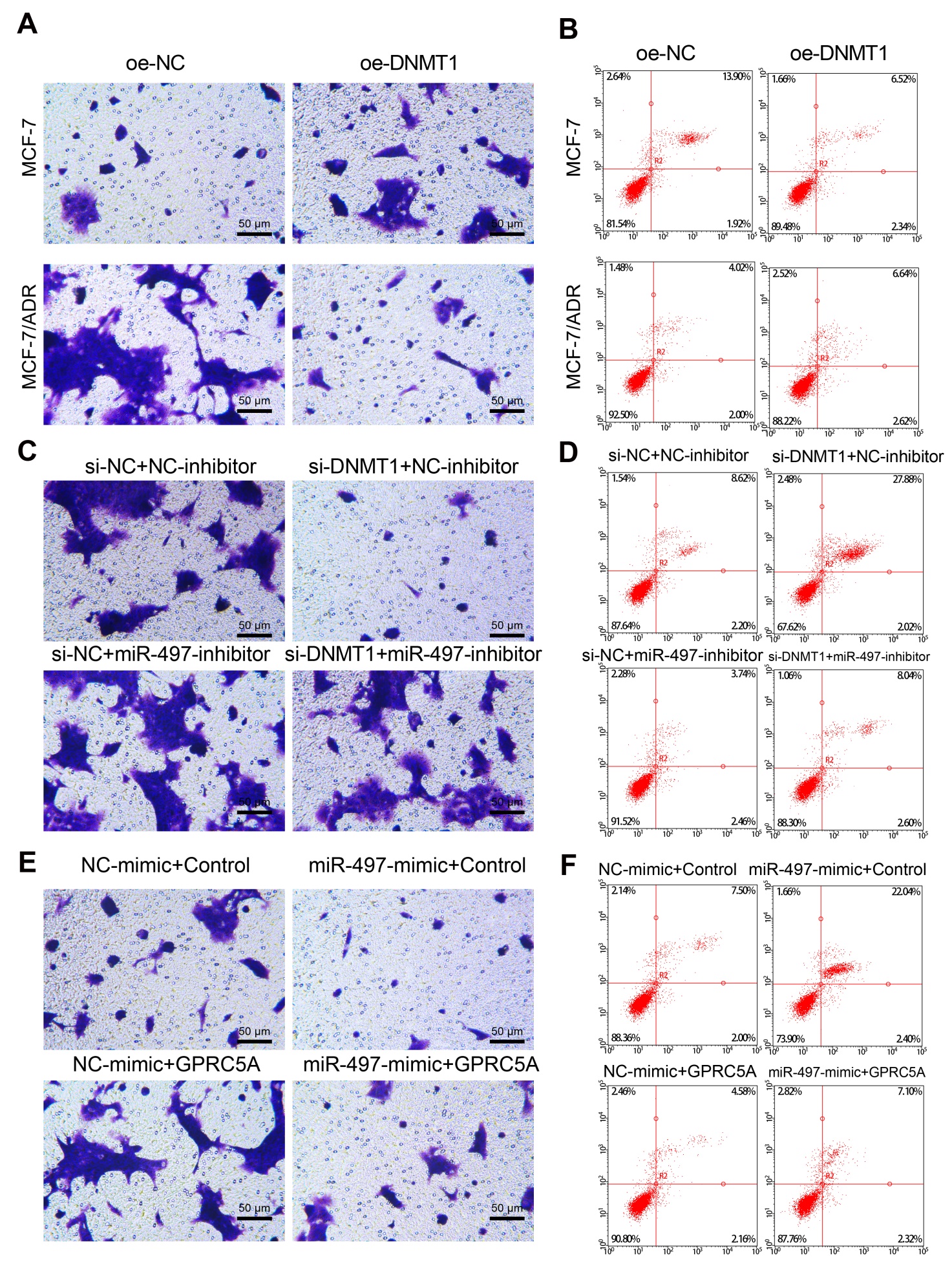
**

**Figure S1.** Representative images for Transwell assay and flow cytometry. A-B, Cell invasion and apoptosis in response to oe-DNMT1, as detected by means of Transwell assay (A) and flow cytometry (B). C-D, Cell invasion and apoptosis in response to si-DNMT1 and miR-497 inhibitor alone or in combination, as detected by means of Transwell assay (C) and flow cytometry (D). E-F, Cell invasion and apoptosis in response to miR-497 mimic and GPRC5A alone or in combination, as detected by means of Transwell assay (E) and flow cytometry (F).
